# Treadmill Training with HAL Exoskeleton—A Novel Approach for Symptomatic Therapy in Patients with Limb-Girdle Muscular Dystrophy—Preliminary Study

**DOI:** 10.3389/fnins.2017.00449

**Published:** 2017-08-08

**Authors:** Matthias Sczesny-Kaiser, Rebecca Kowalewski, Thomas A. Schildhauer, Mirko Aach, Oliver Jansen, Dennis Grasmücke, Anne-Katrin Güttsches, Matthias Vorgerd, Martin Tegenthoff

**Affiliations:** ^1^Department of Neurology, BG-University Hospital Bergmannsheil Bochum, Ruhr University Bochum Bochum, Germany; ^2^Department of General and Trauma Surgery, BG-University Hospital Bergmannsheil Bochum, Ruhr University Bochum Bochum, Germany; ^3^Department of Spinal Cord Injury, BG-University Hospital Bergmannsheil Bochum, Ruhr University Bochum Bochum, Germany

**Keywords:** muscular dystrophy, exoskeleton, locomotor training, walking, rehabilitation

## Abstract

**Purpose:** Exoskeletons have been developed for rehabilitation of patients with walking impairment due to neurological disorders. Recent studies have shown that the voluntary-driven exoskeleton HAL® (hybrid assistive limb) can improve walking functions in spinal cord injury and stroke. The aim of this study was to assess safety and effects on walking function of HAL® supported treadmill therapy in patients with limb-girdle muscular dystrophy (LGMD).

**Materials and Methods:** Three LGMD patients received 8 weeks of treadmill training with HAL® 3 times a week. Outcome parameters were 10-meter walk test (10 MWT), 6-minute walk test, and timed-up-and-go test (TUG). Parameters were assessed pre and post training and 6 weeks later (follow-up).

**Results:** All patients completed the therapy without adverse reactions and reported about improvement in endurance. Improvements in outcome parameters after 8 weeks could be demonstrated. Persisting effects were observed after 6 weeks for the 10 MWT and TUG test (follow-up).

**Conclusions:** HAL® treadmill training in LGMD patients can be performed safely and enables an intensive highly repetitive locomotor training. All patients benefitted from this innovative method. Upcoming controlled studies with larger cohorts should prove its effects in different types of LGMD and other myopathies.

## Introduction

Limb girdle muscular dystrophies (LGMD) are rare neuromuscular diseases with an estimated prevalence ranging from 0.43 per 100,000 for subtype 2I (LGMD 2I) and 0.94 per 100,000 for subtype 2A (LGMD 2A; Fanin et al., 2005; Narayanaswami et al., 2014; Thompson and Straub, 2016). They are a pathophysiologically diverse group of degenerative myopathies with the common feature of floppy para- or quadriparesis with proximal pronouncement and muscular atrophy. Basically, LGMDs and divided into two groups due to different modes of inheritance. The LGMD1 group has an autosomal dominant inheritance, whereas LGMD2 are autosomal recessive. Involvement of cardiac and respiratory muscles, joint contractures and extramuscular anomalies occur irregularly depending on the subtype, the underlying genetic defect and affected structural protein (Thompson and Straub, 2016). For example, cardiac involvement is observed often in LGMD 2B whereas has not yet been reported for LGMD 2A, suggesting different pathophysiological mechanisms in both diseases. Different genetic mutations have been identified as the cause of fiber degeneration and strength loss. Intensive genetic and proteomic research over the last 10 years helped to understand the disease and clinical manifestations. Another nomenclature of LGMDs can be done depending on the specific protein function that is defective. So, different pathways and subcellular structures can be involved, e.g., dystrophin-glycoprotein complex, sarcomere, glycosylation, signal transduction, nuclear function, and trafficking. These pathological mechanisms may lead to loss of sarcomere integrity, error in glycosylation of alpha-dystroglycan, defects in muscle repair and dissociation of the sarcomere. For example, LGMD 2A is the most frequent LGMD worldwide. Underlying mutations are located in the Calpain-3-gene that encodes a muscle-specific nonlysosomal protease that is supposed to be mainly involved in disassembly of the sarcomere and muscle cytoskeleton to allow for proper protein turnover during muscle remodeling (Taveau et al., 2003; Kramerova et al., 2007a,b). The clinically manifestations can vary and the phenotypic spectrum is broad; cardiac involvement has not been reported yet and stands in great contrast with other types of LGMD (Kramerova et al., 2007a). In contrast, LGMD 2I is caused by a mutation in the fukutin-related protein gene (*FKRP*) at chromosome 19. It leads to secondary laminin alpha2 deficiency and an abnormal glycosylation of alpha-dystroglycan (Brockington et al., 2001a,b). The clinical spectrum of this subtype ranges from severe and Duchenne-like phenotypes to mild dystrophies with mild elevated creatine kinase (CK) levels (Mercuri et al., 2003). However, genetic and protein defects for all subtypes are still not yet known. Today, LGMD1 A–H and LGMD2 A–W are described (Nigro and Savarese, 2014) today. Clinically, muscle weakness is usually slowly progressive over years. Resultant reduced endurance and cardiovascular fitness, fatigue, exercise intolerance and a more sedentary lifestyle are common symptoms of patients with LGMD. Sooner or later but depending on subtype, most patients need physical assistance for walking and activities of daily living.

Inspite of growing and innovative pharmaceutical investigations for degenerative and even hereditary neurological disorders, disease-specific therapy for LGMD is not yet available (Thompson and Straub, 2016). A few study groups still try to find therapies that start at genetic level. Initial laboratory approaches have been undertaken for precise genetic therapy in LGMD 2B and 2D (Turan et al., 2016). Other study groups have tried to diminish secondary effects of muscle degeneration like inflammation with immune suppressive therapies or to prevent cardiac involvement (Chen et al., 2016; Heydemann, 2016). None of these approaches are established and safe medical therapies, today. Nonetheless, patients suffering from LGMD cannot profit from these new investigations. Therefore, exercise therapies play an important role in patients with LGMD (Siciliano et al., 2015).

Even many studies have been conducted in this area, little is known about the best and most effective physiotherapy for each different myopathy. Standard rehabilitation therapy programs could not be established yet. Most studies struggle with the problem of small case numbers, unclear subtype of LGMD, unclear genetic mutation and heterogeneous causes of myopathy that has been included to the study. One should be very careful in extrapolating training effects from one disease to another, since the molecular defects are so different, which warrants many more investigations of individual muscle diseases.

Generally, two different types for physical therapy can be divided: (a) strength training and (b) aerobic exercise training (Siciliano et al., 2015). **Strength training programs** aiming at certain target muscles report transient increase in muscle strength, but without knowing the optimal resistance needed (Lindeman et al., 1995; Sveen et al., 2013). Strength exercise should be supervised because of possible long-lasting myalgia and increase of (CK) indicating therapy-induced muscular injury (da Luz et al., 2011). Studies examining various exercise protocols are limited because of small sample sizes and wide clinical heterogeneity, again. Several studies showed that walking rehabilitation programs with **aerobic endurance training** can improve muscle strength and self-assessed muscle function (Ansved, 2003; Vissing et al., 2014). For example, Vissing et al. investigated 6 patients with LGMD 2L, so called anoctamin 5 myopathy (Vissing et al., 2014). They performed systematically aerobic training with a bicycle ergometer for 10 weeks. The authors could show improvement of oxidative capacity and muscle strength without further increase of CK or muscle soreness. Further studies from the same scientific group have demonstrated similar positive results for aerobic training in Becker's dystrophy (Sveen et al., 2008), LGMD 2I (Sveen et al., 2007), and facioscapulohumeral muscular dystrophy (Olsen et al., 2005). However, aerobic training seems to play a useful and safe effect in some subtypes of LGMD.

Today, treadmill training (TT) is a very useful tool to enhance locomotor function in diseases presenting with walking disturbance, e.g., stroke and spinal cord injury (SCI; Protas et al., 2001; Srivastava et al., 2016). It requires partially preserved stability of the trunk and residual active muscle innervation of the lower extremities. Combined with a body weight support (BWSTT), it is a regularly used and safe tool to applicate an aerobic endurance training. BWSTT can be assessed also in patients with impaired cardiac and pulmonary function. Its advantage is a very high repetition frequency of gait cycle that can lead to more intensive learning and training effect (Daly and Ruff, 2007). BWSTT can especially improve the stance phase of gait, i.e., the symmetrical weight shift (Visintin and Barbeau, 1994), symmetrical activation of the quadriceps muscles during limb loading (Trueblood, 2001) and faster walking (Visintin et al., 1998; Daly and Ruff, 2004, 2007). Because BWSTT has some lacks of results in some gait training studies considering brain plasticity and for the swing phase of gait, it is reasonable to combine BWSTT with other interventions. Daly et al. combined BWSTT with functional electrical stimulation (FES) in stroke survivors. They showed that the combination offered the capability of practice of the greatest number of gait components for which two motor learning principles associated with CNS plasticity could be satisfied: practice of close-to-normal movement and repetition of that practice (Daly and Ruff, 2007). Some studies using BWSTT/TT in myopathies can be found in literature. In particular, Taivassalo used TT in patients with metabolic and non-metabolic myopathies performing a short-term aerobic training at low intensity. Both groups benefited (Taivassalo et al., 1998, 1999). Controlled studies aiming on the effect of BWSTT/TT compared to other training methods on different types of myopathy are not available so far. Clear recommendations on BWSTT/TT application and other types of training methods in myopathies do not exist today.

In the last decades, BWSTT has been combined with neurorobotic devices also (Blaya and Herr, 2004; Pohl et al., 2007; Michmizos et al., 2015). All of these devices provide repetitive, accurate and reproducible practice and represent task-specific training. A new generation of robotics are exoskeletons. They are wearable mobile machines with a frame and actuators on hip and knee joints. One commercially available exoskeleton is the hybrid assistive limb® (HAL®; Kawamoto et al., 2013, 2014). It detects electromyographic signals via surface electrodes from the ventral and dorsal thigh (Cyberdyne Inc., Japan). In contrast to other robotic devices that provide automatic passive motion, HAL® enables gait training with voluntarily driven by muscle activity. Today, it used as a training tool only, not as an aid for domestic field. In studies, it used in combination with BWSTT throughout. Studies on patients with SCI and stroke have demonstrated beneficial effects on walking function and on neuronal plasticity (Kubota et al., 2013; Aach et al., 2014; Cruciger et al., 2014; Sczesny-Kaiser et al., 2015). Aach et al. showed in a pilot study and in a larger study including 55 patients with chronic SCI that BWSTT with HAL® led to functional improvement assessed by walking tests like 10-meter walk test and 6-minutes walk test. The authors used an intensive training program with 5 sessions per week over 12 weeks. An improvement of ~50% could be expected (Aach et al., 2014; Grasmücke et al., 2017). Even a cortical reorganization indicating neuronal plasticity could be observed. Using electrophysiological methods, unused leg representations in the postcentral gyrus were activated even after years post ictus (Sczesny-Kaiser et al., 2015). Similar results have been reported for chronic stroke patients (Yoshimoto et al., 2016). Walking speed increased about 56%. A comparison with other rehabilitation programs like Bobath has not been performed so far. Past experience with HAL® training in patients with chronic gait impairment demonstrated that this exoskeleton and training program can induce additional functional improvements. Because patients with chronic myopathy rely on physiotherapy only, HAL® might be a new training tool for exercise training. Looking for HAL® training in myopathies, only one case has been reported using HAL® in a patient with ocularpharyngodistal myopathy (Hasegawa et al., 2015). Authors stated that no adverse events occurred and that patient's walking parameters were stable. Further application of HAL®-training in myopathies or muscular dystrophies does not exist. Because HAL® exoskeleton is a promising and novel rehabilitation tool utilizing patients' active and voluntarily driven muscular activity, we evaluated the effect on walking functions in three LGMD patients before and after an 8-week period of HAL®-supported treadmill training. By means of the results of this small study, upcoming studies with larger groups should be planned. We want to establish a novel and innovative therapy program for patients with myopathies.

## Materials and methods

### Subjects

Three outpatients of our neuromuscular center with LGMD were enrolled. Clinical details are shown in Tables 1A,B. Inclusion criteria were a clinically, histopathologically, or genetically determined LGMD with para- or quadriparesis and proximal pronouncement and muscular atrophy, and sufficient stability of the trunk. The patients were required to present preserved motor function of hip and knee extensor and flexor muscle groups in order to be able to trigger the exoskeleton (medical research council scale for muscle strength = MRC ≥ 1/5). Exclusion criteria were plegia (MRC 0/5), instability of the trunk, severe limitations of range of motion regarding hip and knee joints (i.e., leading to functional contractures), body weight >100 kg, non-consolidated fractures and severe heart insufficiency. All patients provided written informed consent (ethic approval no. 4733-13, Medical faculty, Ruhr University Bochum). The study was performed in accordance with the Declaration of Helsinki. The clinical assessment and muscle strength score were evaluated before the protocol by a skilled operator.

**Table 1:** A and B: Clinical data of LGMD patients.

**A**				
**No.**	**Age range**	**Diagnosis**	**Genetics**	
1	40-45	LGMD 2A	Compound heterozygous *CAPN3*-mutations in exon 3 c.390G>C (p.Trp130Cys) and in exon 4: c.550delA	
2	55-60	LGMD 2I	Homozygous *FKRP*-mutation in exon 4: c.826C>A (p.Leu276Ile)	
3	60-65	LGMD (of unknown subtype)	No mutations detected in next generation sequencing panel including *CAPN3*-, *DYSF*-, *FKRP*-genes	
**B**				
**No**.	**Age range at disease onset**	**Proximal muscle strength of lower extremities (MRC)**	**Extraskeletal muscular involvement**	**Physical assistance**
1	10–15	1–2/5	None	Electric wheelchair
2	45–50	2–3/5	Cardiac, mild lowered left ventricular function	Wheeled walker
3	45–50	2–3/5	None	Walking stick

*M, man; F, female; LGMD, limb-girdle muscular dystrophy; MRC, medical research council*.

### The exoskeletal training

**Figure 1:**
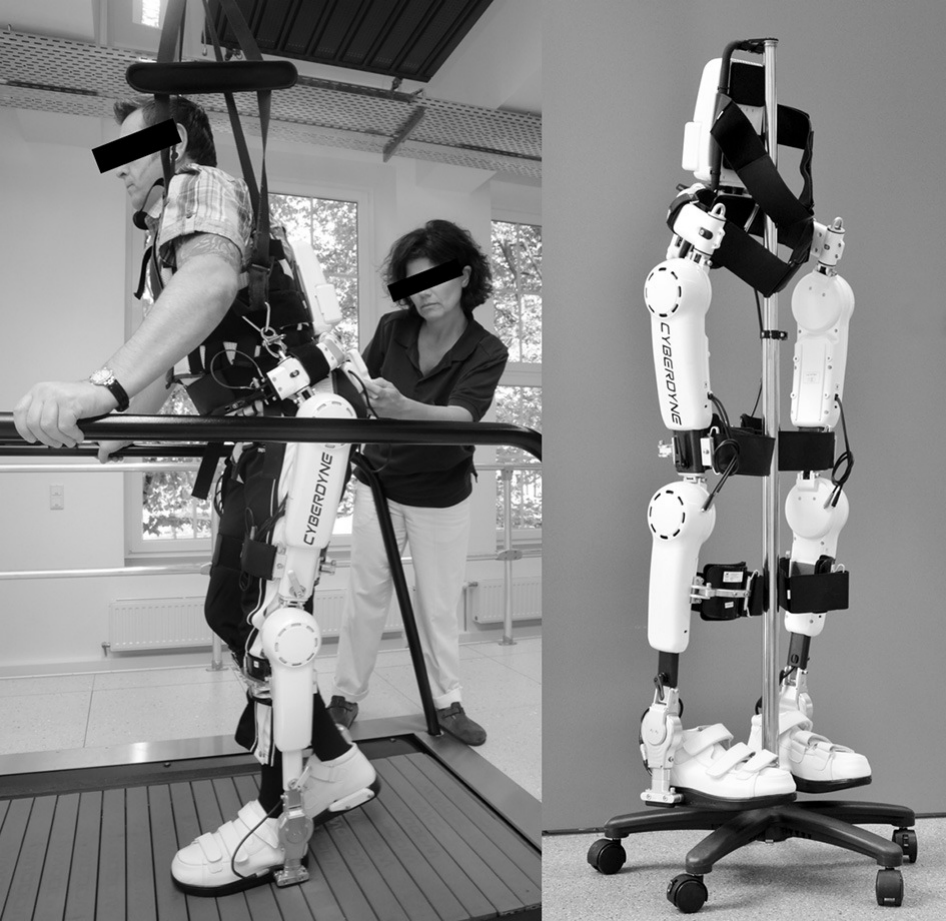
Training setting. The patient is performing body-weight supported treadmill training with the HAL® exoskeleton. The physiotherapist supervises the session. Left picture: Copyright V. Daum, Bergmannsheil Bochum; with informed and written consent obtained from both, the patient and the therapist; right picture: Copyright and courtesy of Cyberdyne Care Robotics GmbH, Bochum, Germany. Appropriate permissions have been obtained from the copyright holders for the publication of both images.

All patients underwent an 8-week training period of body-weight supported treadmill training (BWSTT) with HAL® exoskeleton. Each patient was scheduled 3 times a week resulting in 24 sessions. The actual time of walking with HAL® was max. 30 min. The exoskeleton was voluntarily triggered by EMG-signals from the extensor and flexor muscles of hip and knee detected via surface EMG electrodes. Voluntary motion was magnified and adjusted the muscle activity in accordance with the patient's intention. During therapy, the velocity of the treadmill (Woodway USA, WI, USA) was set individually between comfortable and maximum speed tolerated by the patient. Initially, the bodyweight support was individually pre-set at up to 50% of patient's body weight and individually reduced in subsequent training sessions (see Figure 1).

### HAL^®^-supported treadmill parameters

Some treadmill-bounded parameters were assessed. The distance on treadmill in meters for each session, the velocity on treadmill and the time on treadmill were recorded and documented by the therapist.

### Primary outcome measurements

**Figure 2:**
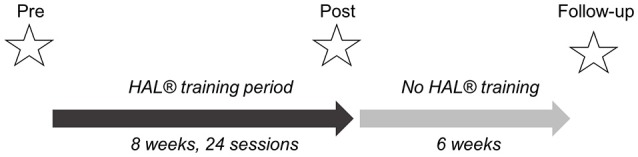
Study design. Three measurements: pre-, post-, and follow-up assessments. Additionally, 10 MWT was performed before and after each training session. Follow-up assessments were done 6 weeks after completion of HAL® training period.

To assess the training effect, all tests were performed *without* HAL® exoskeleton. We used the 10-meter walk test (10 MWT) at each daily training session (Bohannon et al., 1996). Data from the first and the last day have been taken for baseline and end of the training data. We took it daily in order to give a feedback to the patient, to motivate her/him. The 10 MWT measured the time needed to walk a 10 m distance. The timed-up-and-go test (TUG) describes the time and assistance required for standing up from the wheelchair, walk 3 m, turn around, walk back and sit down (Podsiadlo and Richardson, 1991). The 6-minute walk test (6 MWT) evaluates the distance covered when walking for 6 min (Balke, 1963). TUG test and 6 MWT were done at the beginning and at the end of the 8-week training period. If possible, all three tests were assessed 6 weeks after the end of the training period again (follow-up assessment, see Figure 2).

### Secondary outcome measurements

To assess motor and balance functions, the motor-related section of the Functional Independence Measure (FIM) (Keith et al., 1987) and the Berg Balance Scale (BBS) (Berg et al., 1992) were documented at baseline and after the 8-week training period (Ottenbacher et al., 1996; Ravaud et al., 1999; Bérard et al., 2005). Both scales were tested by a physical therapist with long-time experience in neurological disorders and myopathies.

### Self-reported changes in condition and symptoms, adverse events

Before and after each training session, patients were asked if they had any negative symptoms and adverse events. Furthermore, after the whole training session, patients completed a modified questionnaire grading changes in physical endurance, leg muscle strength, and physical activity (Sveen et al., 2007).

## Results

### Exoskeletal training

All three patients completed 24 sessions each. Patients #2 and #3 performed follow-up assessment after 6 weeks. Patient #1 was lost to follow-up. Adverse reactions did not occur. Assessed data can be divided into two categories: (a) HAL®-supported treadmill parameters and (b) primary outcome parameters *without* HAL®. Treadmill parameters were recorded online and demonstrate the velocity on treadmill, how long the patients could perform the exercise on the treadmill and the distance walked on treadmill. These parameters should improve, otherwise the training has to be considered not suitable for this patient. Improvement in treadmill bounded parameters are the basis of a successful training.

### HAL^®^-supported treadmill parameters

**Figure 3:**
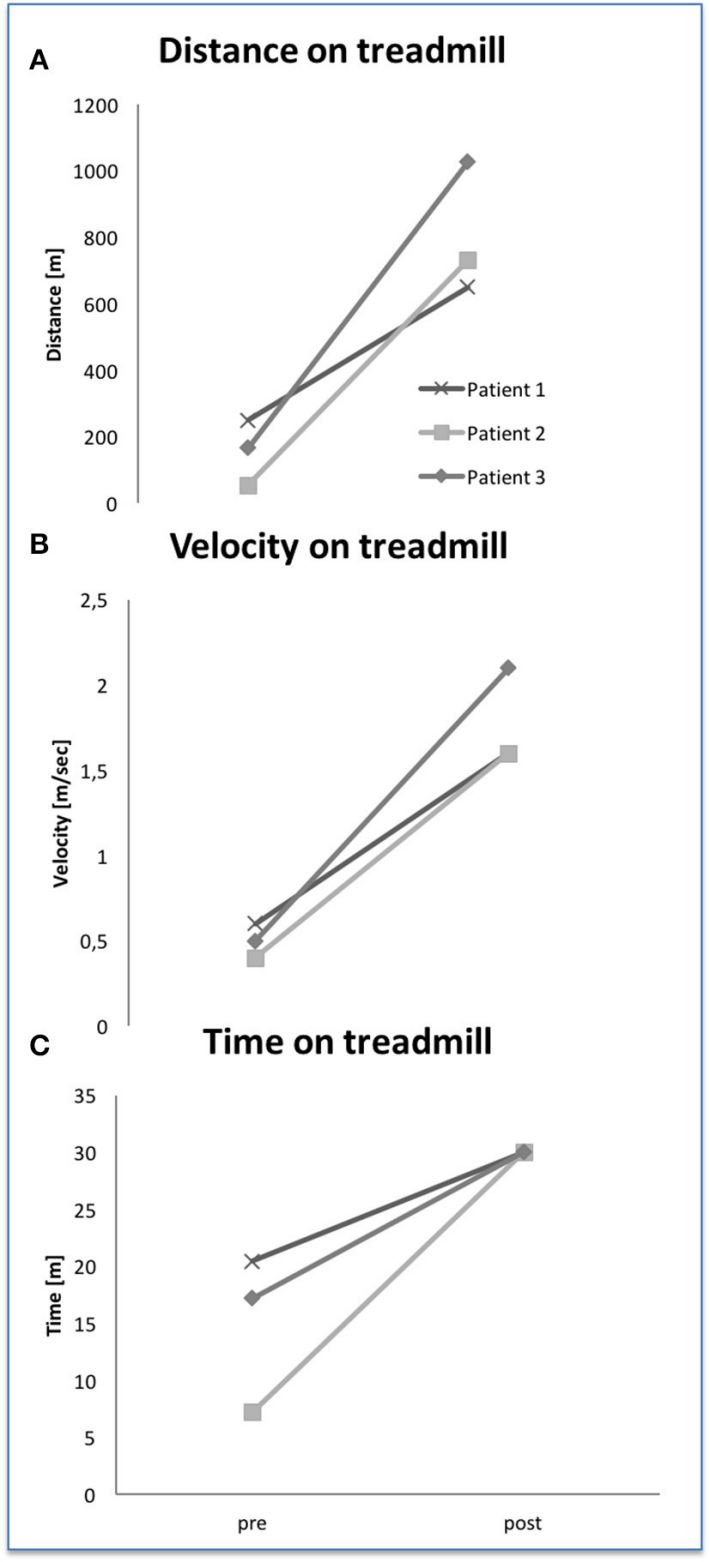
Treadmill parameter with HAL® exoskeleton at baseline and after 24 training sessions **(A–C)**.

All patients showed increased distance covering on treadmill, increased training time with a maximum of 30 min and increased velocity on treadmill (see Figures 3A–C). A five-fold distance, four-fold increase of velocity and a maximum time on treadmill (30 min) have been reached in all patients.

### Primary outcome parameters

**Figure 4:**
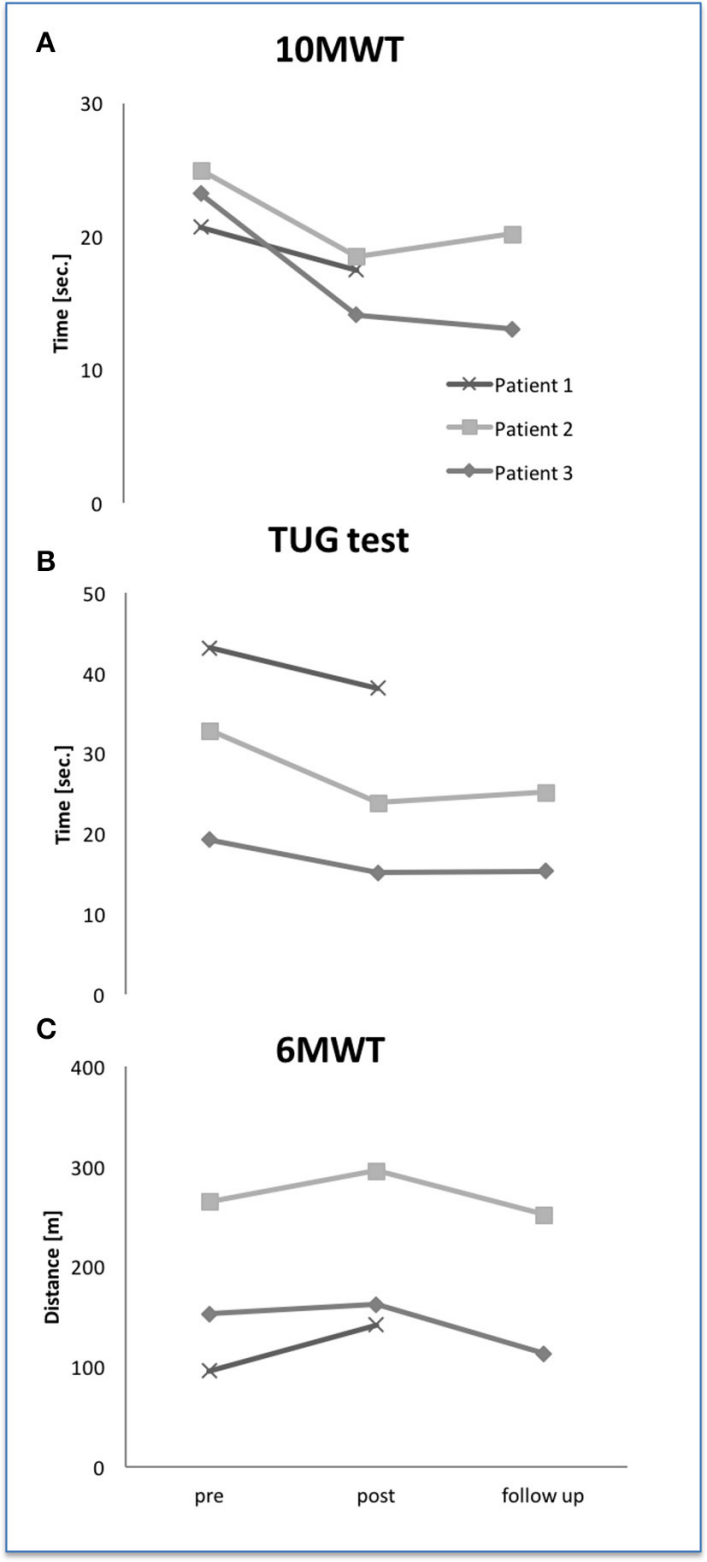
Walking abilities at baseline, after 24 HAL® training sessions and follow up 6 weeks later. Ten MWT, 10-meter walk test **(A)**; TUG, timed-up-and-go **(B)**; 6 MWT, 6-minute walk test **(C)**.

Figures 4A–C show the results of all walking parameters 10 MWT, TUG test, and 6 MWT. At baseline 10 MWT showed similar impairment at a mild stage for all 3 patients. Six MWT and TUG test demonstrated different degree of impairment reflecting different subtypes of LGMD. But, assessed *without* HAL® exoskeleton, all patients showed clearly decreased time in 10 MWT, increased distance in 6 MWT and decreased time in TUG test after 24 training sessions. In the follow up assessments, 10 MWT and TUG test data revealed preserved training effects in both patients compared to baseline data. This effect could not be observed for 6 MWT data. Statistical analysis was not performed due to small data.

### Secondary outcome measurements

**Figure 5:**
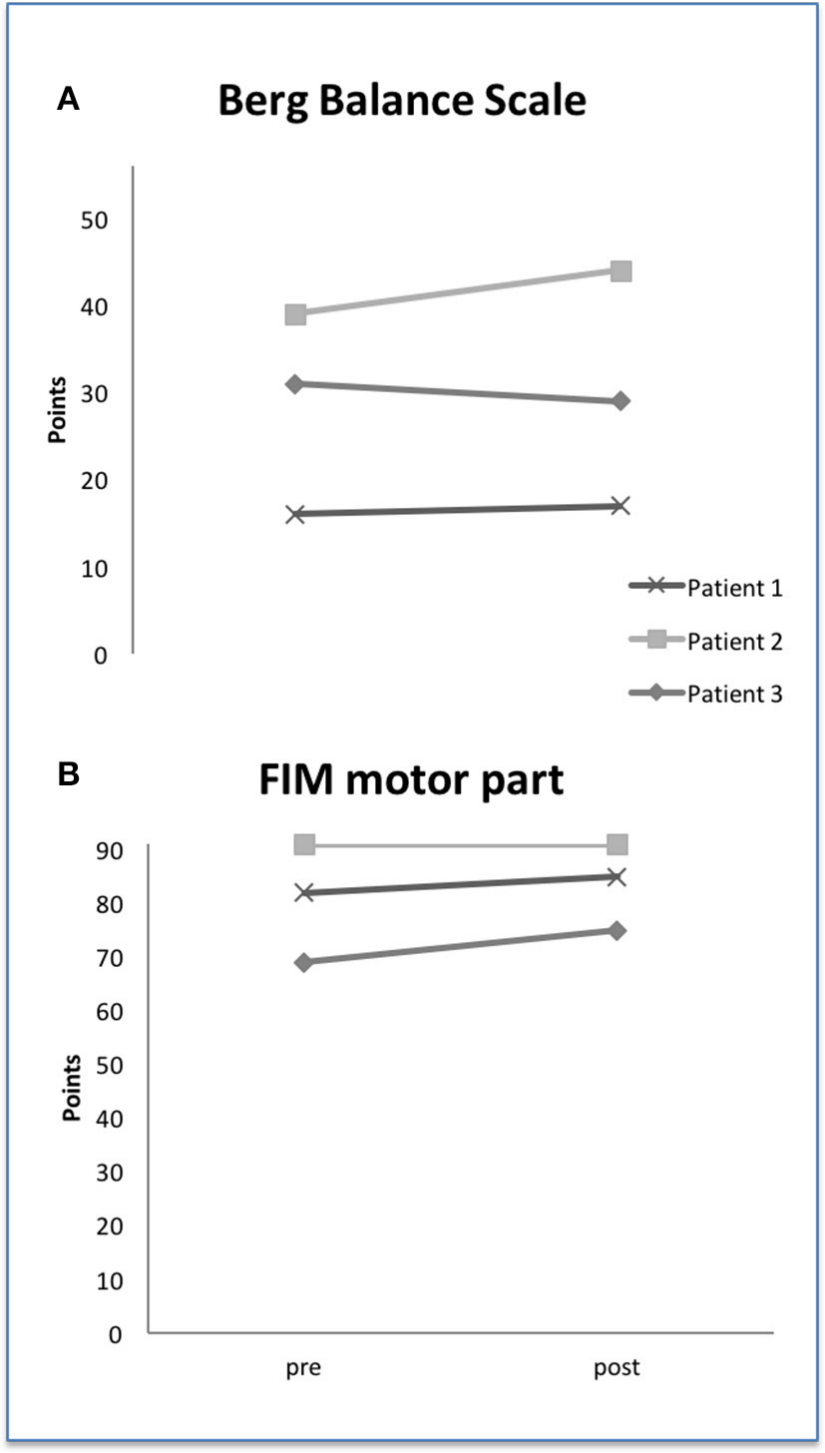
Berg-Balance Scale **(A)** and FIM motor part **(B)** at baseline and after HAL® training. FIM, functional independence measure.

Patient #2 showed increased BBS-score of 5 points after training period. BBS-scores of patients #1 and #3 varied only by 1 point. Considering the motor section of FIM, patient #3 increased by 6 points after HAL® therapy whereas patient #2 did not exhibit any change (see Figures 5A,B). Patient #1 could increase his performance by 3 points.

### Self-reported improvements and adverse events

Patient #1 and #2 did not report any adverse events. Patient #3 felt general weakness for about 1–2 h after the training session without myalgia or stiff muscles. Furthermore, all three patients told about better physical endurance after 8 weeks of HAL® training (see Table 2).

**Table 2:** Self-reported training-induced changes of physical fatique, endurance, muscle strength and walking distance.

**Patient**	**Physical fatique**	**Physical endurance**	**Leg muscle strength**	**Walking distance**
#1	0	+	0	0
#2	+	+	+	+
#3	0	+	0	+

*+, improvement; 0, no change; −, worsening*.

## Discussion

This study shows that HAL®-assisted BWSTT is a safe and effective method for aerobic exercise training in ambulatory patients with LGMD. All patients showed increased *treadmill-bounded* and HAL®-*dependent* walking parameter, i.e., walking distance, velocity on treadmill and time on treadmill. No patient reported about sustained worsening of symptoms. These results indicate a good acceptance of this new robotic training method and give the basic for an effective therapy. HAL® supports the patients efficiently to complete treadmill training sessions and to improve walking distances on treadmill. HAL®-training enables a highly repetitive and intensive locomotor training in LGMD-patients with mild to severe impaired walking functions. Like previously described, TT cannot cover all parts of gait phases; TT is more effective when combined with other training methods. In our study, we combined with a voluntarily driven exoskeleton. In this combination, more importantly, we found improved HAL®-*independent* walking parameters (10 MWT, 6 MWT, TUG) which were paralleled by self-reported improvements. Compared to other HAL® BWSTT studies, we found similar improvements with ~30–50% for walking speed. The effects on TUG test were in two patients (#1, #3) smaller; patient #2 clearly improved in TUG test ~30%. Again, we can see different effects on different types of myopathy. The effects on 6 MWT were smaller. These results indicate different effects of HAL®-supported BWSTT on different aspects of gait, too. Ten MWT mainly reflects walking speed and an efficient sequence of gait phases whereas TUG test aims on safety, balance and functional mobility. Six MWT includes cardiopulmonary fitness. Parameter of pulmonary and cardiac functions were not assessed in this study. One might speculate only about the possible reason for the lack of improvement in 6 MWT. A comparison with aerobic exercise studies on LGMD patients assessed by Sveen (Sveen et al., 2007) and Vissing (Vissing et al., 2014) is not available. Sveen and Vissing used parameters for cardiopulmonary fitness like V_O2_ and plasma lactate levels. These parameters were not collected in our study. Taivassalos' studies on metabolic and non-metabolic myopathies using TT assessed different parameters, too phosphorus magnetic resonance spectroscopy (Taivassalo et al., 1998, 1999). Thus, our study results cannot be compared with previous studies dealing in this field.

Remarkably, training effects measured with 10 MWT and TUG persisted for at least 6 weeks. The effect on 6 MWT could not be detected at follow-up. The reason for this observation remains unclear. Maybe a short-time factor like motivation might play a role. Missing effects on cardiopulmonary functions might be another reason. Since cardiopulmonary parameters like V_O2_ were not revealed, this question remains unanswered.

In order to answer the question whether HAL® training effects are limited to walking function or can also lead to better motor and balance functions in daily living, FIM and BBS were assessed. The results of both parameters were inconsistent and have to be tested in a larger cohort. The observed score changes are small and do not exceed the required minimal detectable change. One might see a tendency for patient #2 in BBS. The patient also improved in TUG test that includes balance functions also. So, both values (BBS and TUG test) might be considered as a concordant result. Patient #2 reported about frequent falls that reduced after training period for a time. Whether this can be a systematic result, upcoming studies have to investigate this issue.

This is the first HAL® study specifically focused on patients with LGMD. Patients with this disease present with proximal paresis. Because HAL® robot suit supports proximal joints and muscle groups, these patients might benefit the most from this therapy. As mentioned above, two single case reports have been published including one distal and one proximal pronounced myopathy (Asai et al., 2014; Hasegawa et al., 2015). Like the patient with BMD and proximal pronounced paresis, all our LGMD patients improved in HAL®-independent walking parameters. In contrast to this, the OPDM patient's walking function did not improve whereas treadmill associated parameters increased. These two single cases underline that HAL® training is more effective in patients with proximal paresis. So, from our point of view, it is necessary to adapt HAL®-training and take into account the physical condition and pattern of paresis. Another critical point is the training frequency and training time. The whole training session lasted about 90 min, the net walking time 30 min. Coming up from our previous SCI study and to take possible myalgias into account, we reduced the frequency from 5 times a week to 3 times a week (Aach et al., 2014; Nilsson et al., 2014). The question which frequency might be optimal should be tested in upcoming systematic studies.

Considering that these patients suffer from chronic degenerative and dystrophic myopathy, these results are encouraging, offering the possibility to use a novel approach of symptomatic therapy. Even though our study does not allow conclusions on interference with natural course of LGMD or potential long-term effects as compared to conventional physiotherapy, the results are encouraging for upcoming controlled HAL®-studies in larger groups of patients with muscular dystrophy. The anatomical or pathophysiological origins of positive effects on walking functions remain unclear. Different effects on muscular and neuronal systems can be discussed but were not investigated in detail in this study.

Instead of our positive results, some limitations should be discussed. This study is based on only three LGMD patients without a control group. All patients were ambulatory patients of our neuromuscular center and are not representative of the disease with respect to age, gender, and clinical aspect, thus the findings cannot be generalized to the whole LGMD population. Specific measurements looking for increased endurance capacity (lactate, respiratory function, cardiovascular measurements) and serum CK levels were not collected. In future studies, these parameters should be implemented.

We can conclude that our study investigating HAL®-assisted body-weight supported treadmill training in patients with LGMD showed feasibility and safety. Moreover, for the first time, our data show that this voluntarily driven exoskeleton can improve walking functions. With respect to the limited data of three patients only, it encourages us to undertake further studies with larger cohorts and different types of LGMD.

## Author contributions

MS, RK, OJ, DG had substantial contributions to the conception and design of the work, acquired data and analyzed the data, drafted the work, and finally approved the final version to be published. MA, AG, MV, TS, and MT participated in the coordination, the design and drafted the manuscript and made critical revisions of the manuscript.

### Conflict of interest statement

MS, RK, MA, OJ, DG, AG, MV, and MT declare that the research was conducted in the absence of any commercial or financial relationships that could be construed as a potential conflict of interest. TS reports personal fees from Cyberdyne, Inc. outside the submitted work.
